# Validation of a Predictive Model for Survival in Patients With Advanced Cancer: Secondary Analysis of RTOG 9714

**DOI:** 10.4021/wjon325w

**Published:** 2011-08-24

**Authors:** Edward Chow, Jennifer L. James, William Hartsell, Charles W. Scarantino, Robert Ivker, Mack Roach, III, John H. Suh, William Demas, Andre Konski, Deborah Watkins Bruner

**Affiliations:** a Odette Cancer Center, Toronto, ON, Canada; b RTOG Statistical Center, Philadelphia, PA, USA; c Good Samaritan Cancer Center, Downers Grove, IL, USA; d Rex Healthcare Cancer Center, Raleigh, NC, USA; e Newark Beth Israel Medical Center, Newark, NJ, USA; f University of California San Francisco, San Francisco, CA, USA; g Cleveland Clinic Foundation, Cleveland, OH, USA; h Akron City Hospital, Akron, OH, USA; i Fox Chase Cancer Center, Philadelphia, PA, USA; j University of Pennsylvania School of Nursing, Philadelphia, PA, USA

**Keywords:** Survival prediction, Advanced cancer

## Abstract

**Background:**

The objective of this study was to validate a simple predictive model for survival of patients with advanced cancer.

**Methods:**

Previous studies with training and validation datasets developed a model predicting survival of patients referred for palliative radiotherapy using three readily available factors: primary cancer site, site of metastases and Karnofsky performance score (KPS). This predictive model was used in the current study, where each factor was assigned a value proportional to its prognostic weight and the sum of the weighted scores for each patient was survival prediction score (SPS). Patients were also classified according to their number of risk factors (NRF). Three risk groups were established. The Radiation Therapy and Oncology Group (RTOG) 9714 data was used to provide an additional external validation set comprised of patients treated among multiple institutions with appropriate statistical tests.

**Results:**

The RTOG external validation set comprised of 908 patients treated at 66 different radiation facilities from 1998 to 2002. The SPS method classified all patients into the low-risk group. Based on the NRF, two distinct risk groups with significantly different survival estimates were identified. The ability to predict survival was similar to that of the training and previous validation datasets for both the SPS and NRF methods.

**Conclusions:**

The three variable NRF model is preferred because of its relative simplicity.

## Introduction

Survival prediction of patients with advanced cancer is one of the most difficult and least welcomed tasks clinicians have to face. However such an estimate is very important in end-of-life care [1]. Inaccurate prediction of survival often results in inadequate access to palliative care. Overly optimistic prediction may deter patients from being involved in palliative programs at an earlier stage.

Patient preferences about the trade-off between the risks and benefits associated with treatment strategies are often based on perceptions of prognosis. Inaccurate perceptions can lead to unrealistic expectations [2]. Weeks and colleagues found that patients with a misperceived optimistic prognosis often request medical therapies that most physicians would consider futile. These same patients were 8.5 times more likely to favor receiving aggressive, life-extending medical care than were patients with more accurate estimates of their 6-month survival. More disturbingly, those with overly optimistic prognoses were more likely to die in hospital on mechanical ventilation than were those patients with more realistic estimates of their survival potential. The authors conclude that terminal cancer patients’ miscalibrated optimistic prognosis may lead them to choose highly aggressive, invasive and ultimately futile medical care rather than palliative care [3].

The accurate classification of patients with advanced cancer into groups with similar and predictable survival has the potential to improve delivery of care and minimize undertreatment or overtreatment [2, 4]. In validating a classification model, there is a hierarchy of increasing stringent validation strategies [5]: 1) Internal – evaluation restricted to a single training data set; 2) Temporal – evaluation on a second data set at a different time point from the same center; 3) External – evaluation on data from a different center, perhaps by different investigators.

We previously developed a predictive model for patients with advanced cancer by employing three readily available parameters: primary cancer site, site of metastases and Karnofsky performance score (KPS). The initial classification model was developed with a training data set (n = 395) comprised of patients treated at Sunnybrook Odette Cancer Center’s Rapid Response Radiotherapy Program (RRRP) in 1999. Three risk groups–low, intermediate, high–were determined by partial score method and number of risk factors (NRF) method (details in materials and methods section) (Table 1) [6, 7]. A temporal validation set (n = 445) of patients treated in the RRRP in 2000 and an external validation set (n = 467) of patients treated at Princess Margaret Hospital’s Palliative Radiation Oncology Program in 2002 were used to successfully evaluate the initial model.

**Table 1:** Risk Groups Previously Identified

Prognostic Factor	Partial Score Method	Risk Factor Score Method
Primary tumor site		
Breast	0	0
Prostate	2	1
Lung	3	1
Others	3	1
Site of metastases		
Bone only	0	0
Other	2	1
KPS		
> 60	0	0
≤ 60	3	1
	Survival Prediction Score (SPS) determined by sum of partial scores	Number of Risk Factors Score (NRF) determined by sum of risk factors
	Risk Group A [SPS 0 - 4]	Risk Group I [NRF 0 - 1]
	Risk Group B [SPS 5]	Risk Group II [NRF 2]
	Risk Group C [SPS 6 - 8]	Risk Group III [NRF 3]

Note: Sum of partial scores equal to survival prediction score (range 0 to 8).

KPS: Karnofsky performance score

The objective of this secondary study was to use Radiation Therapy Oncology Group (RTOG) 9714 data to provide an additional external validation set to the two methods comprised of patients treated among multiple institutions in the United States and Canada.

## Materials and Methods

### Patient population

The Radiation Therapy and Oncology Group (RTOG) and the North Central Cancer Treatment Group conducted a randomized Phase III trial with breast or prostate cancer patients with bone metastases (RTOG 9714) [8]. Eligible patients had moderate or severe pain, as indicated by a Brief Pain Inventory (BPI) worst pain score of 5 and above or narcotic medication with a daily oral morphine equivalent dose of at least 60 mg if pain scores were < 5. Patients were randomized between treatment with a single fraction of 8 Gy and 30 Gy in 10 fractions. The Karnofsky performance status of the enrolled patients was at least 40, with an estimated life expectancy of at least 3 months. Patients were excluded if there was prior radiation therapy or palliative surgery to the planned radiation treatment site, pathologic or impending fracture, or compression of the spinal cord or cauda equina [8]. The survival status and the date of death from any cause or last follow up of the patients were current as of February 2009.

### Predictive models

#### Partial score method (SPS)

A prognostic score (partial score) based on the regression coefficients of the Cox regression model was assigned to the three factors [primary cancer site (breast/prostate/lung/others), site of metastases (bone/others), and KPS (> 60/≤ 60)] as in the previous work (Table 1) [6]. The survival predictive score (SPS) for a given patient was obtained by adding together his/her partial scores for the three factors [6, 9]. Patients were classified into risk groups based on their SPS score. Three risk groups A, B and C were established.

#### Number of risk factors method (NRF)

The patients were also grouped according to the total number of risk factors (NRF) that they possessed [6]. The three risk factors are as follows: (1) non-breast (i.e., prostate), (2) site of metastases other than bone only, and (3) KPS ≤ 60. Three risk groups I, II and III were established.

#### Comparison of risk groups

The logrank test was used to determine differences in overall survival in the resultant risk groups. The resultant median survival estimates in each risk group were also compared to those of the corresponding risk group in the previous datasets for similarity. Additional methods were then used to evaluate model discrimination. The C index of predictive discrimination measures the proportion of correct predictions based on observed responses. The C index ranges from 0 to 1 with values of 0.5 indicating no predictive discrimination (random classification) between patients with different outcomes and values close to 1 indicating perfect discrimination (proper classification) [10]. The Royston and Sauerbrei D statistic is also a measure of discrimination of the survival model based on its ability to separate survival estimates between risk groups. Higher values indicate a better degree of model separation [11].

## Results

The RTOG external validation set comprised of 908 patients treated at 66 different radiation facilities from 1998 to 2002. Canadian patients represented only 4% of the patient population (Table 2). The mean age was 65 years (SD 12). There were slightly more females (51% vs. 49%). Although geographic variability was obtained, RTOG 9714 had a fairly homogenous population in regards to prognostic variables. All patients had bone metastases and had either a breast (51%) or a prostate (49%) primary tumor, as these disease specifications were trial eligibility criteria. Only twenty-four percent of patients had KPS ≤ 60 as a life expectancy of at least 3 months was also a trial eligibility criterion [8].

**Table 2:** Patient Characteristics From RTOG Validation Set (N = 908)

Characteristic	n (%)
Gender	
Male	447 (49)
Female	461 (51)
Age (years)	
Mean ± SD	65 ± 12
Median (range)	67 (31 - 92)
Country of Residence	
Canada	38 (4%)
USA	870 (96%)
Primary cancer site	
Breast	462 (51)
Prostate	446 (49)
Karnofsky performance status (KPS)	
40 - 60	214 (24)
70 - 100	694 (76)
Physician predicted survival time (months)	
Mean ± SD	14.5 ± 14
Median (range)	12 (1 - 99)
Painful site(s)	
Single	542 (60)
Multiple	366 (40)
Site of radiotherapy	
Weight bearing	506 (56)
Non-weight bearing	402 (44)
BPI worst pain score at study entry*	
< 5 with ≥ 60 mg/day morphine	17 (2)
5 - 6	254 (28)
7 - 10	637 (70)
Receiving biphosphanotes at study entry	
No	688 (76)
Yes	220 (24)
Radiation treatment assignment	
8 Gy	460 (49)
30 Gy	448 (51)

*BPI: Brief Pain Inventory

The SPS method classified all patients into the low-risk Group A with scores from 0 - 4 (Table 3). The 3 month survival estimate for patients in Group A was 84% which was comparable to that of the training set at 82% (Table 4). Given that there were no identified intermediate/high-risk patients, the C index of discrimination was 0.96, indicating near perfect model discrimination.

**Table 3:** Derivation of Risk Groups From RTOG Validation Set

Prognostic Factor	Parameter Estimate*	Standard Error	Hazard Ratio [95% CI]	P-value	Partial Score	Risk Factor Score
Primary Cancer Site						
Breast	0.00		1.00		0	0
Prostate	0.42	0.07	1.52 [1.33, 1.74]	< 0.0001	2	1
						
Site of metastases						
Bone only	—	—	—	—	0	0
						
Karnofsky Performance Status						
> 60	0.00				0	0
≤ 60	0.46	0.08	1.58 [1.35, 1.85]	< 0.0001	2	1
Survival Prediction Score (SPS) [sum of partial scores]					Risk Group A [SPS 0 - 4]	
Number of Risk Factors Score (NRF) [sum of risk factors]					Risk Group I [NRF 0 - 1]	
					Risk Group II [NRF 2]	

*Cox Proportional Hazards Regression Model

**Table 4:** Summary of Model Performance and Survival Estimates: Partial Score Method

Predictive Model	Training Set	Temporal Validation Set	External Validation Set [Single Institution]	RTOG External Validation Set [Multiple Institutions]
	N = 395	N = 445	N = 467	N = 908
Model Performance				
Harrel C index	0.66	0.65	0.63	0.96
Risk Group A				
[Survival Prediction Score, 0 - 4]				
n (%)	108 (27%)	126 (28%)	65 (14%)	908 (100%)
Median Survival (weeks)	60	53	64	42
95% CI	[41, 70]	[36, 75]	[28, undefined]	[38, 45]
Survival Probabilities				
3 months	82%	86%	83%	84%
6 months	70%	72%	64%	66%
9 months	52%	51%	53%	43%

Based on the NRF, two risk groups were identified. Prostate patients with KPS ≤ 60 were classified into the intermediate-risk group II. Eleven percent of patients had those 2 risk factors. Prostate patients with KPS > 60 and all breast patients were classified into the low-risk Group I. Eighty-nine percent of patients had those 0 - 1 risk factors. The 3 month survival estimates for patients in Group I and Group II were 85% and 72%, respectively, which was comparable to that of the training set at 80% and 73%, respectively (Table 5). Given that there were no identified high-risk patients, the C index of discrimination was 0.94, indicating near perfect model discrimination. The Royston and Sauerbrei D statistic was 0.82, indicating good model discrimination.

**Table 5:** Summary of Model Performance and Survival Estimates: Number of Risk Factors Method

Predictive Model	Training Set	Temporal Validation Set	External Validation Set [Single Institution]	RTOG External Validation Set [Multiple Institutions]
	N = 395	N = 445	N = 467	N = 908
Model Performance				
Harrel C index	0.65	0.66	0.63	0.94
Royston and Sauerbrei D statistic	1.09	1.08	0.84	0.82
Risk Group I				
Number of Risk Factors* ≤ 1				
n (%)	98 (25%)	116 (26%)	64 (14%)	812 (89%)
Median Survival (weeks)	60	55	64	45
95% CI	[37, 70]	[37, 91]	[28, undefined]	[41, 51]
Survival Probabilities				
3 months	80%	87%	83%	85%
6 months	68%	73%	63%	69%
9 months	53%	54%	53%	46%
Risk Group II				
Number of Risk Factors = 2				
n (%)	166 (42%)	193 (43%)	189 (40%)	96 (11%)
Median Survival (weeks)	26	19	28	23
95% CI	[20, 31]	[17, 28]	[22, 34]	[17, 31]
Survival Probabilities				
3 months	73%	68%	76%	72%
6 months	51%	45%	52%	46%
9 months	26%	23%	25%	19%

*Risk factors include prostate primary tumor and KPS ≤ 60.

**Figure 1:**
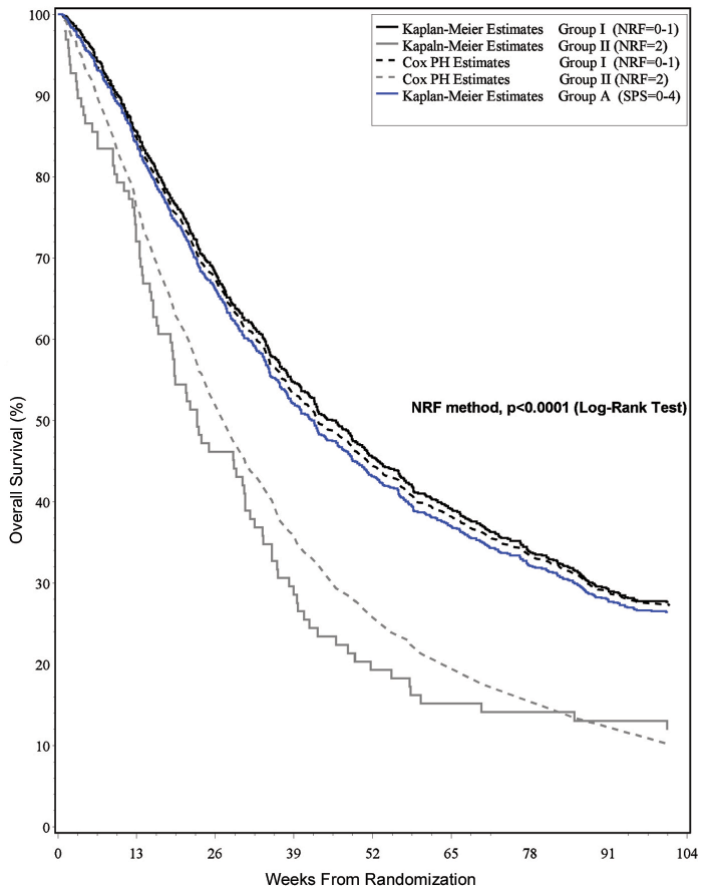
Survival estimates and risk classification group.

Based on these results, the NRF method is preferable to the SPS method in that it gives a more accurate classification of patients and requires simpler calculation. The NRF method is able to further distinguish intermediate-risk patients that the SPS method classifies into a low-risk group only. The difference in survival estimates of the two classification groups was statistically significant (P < 0.0001) (Fig. 1).

## Discussion

Physicians are often requested to predict patient survival at times of referral to hospice programs and enrollment into clinical trials [2]. However, clinicians are often overly optimistic in the survival prediction of terminally ill cancer patients [12, 13]. Parkes, in a commentary, aired his disappointment that doctors are still no better at predicting the length of survival of terminally ill patients than they were 27 years ago. He also stated that if all predictions had been divided by two, they would have been marginally more accurate. He urged that prognoses should be based on proven indexes and not intuition. Physicians need to stop guessing, and when predictions are needed, they should make use of the available predictive instruments [14].

The inaccuracy of survival prediction by palliative radiation oncologists has been well documented. After consultations of cancer patients with metastatic disease for referral of palliative radiotherapy, six radiation oncologists provided survival estimates for 739 patients. These were compared with the actual dates of death obtained from the Cancer Death Registry. The prediction of survival by palliative radiation oncologists was inaccurate and tended to be overly optimistic [15]. Hartsell et al reported the physician prediction of the survival of patients in RTOG 9714. Again the survival prediction was optimistic compared to actual survival by an average of 3 months. The median survival of the 618 expired patients was 6.5 months and the median physician prediction of survival was 12 months [16].

Reviews on survival prediction were conducted by Glare et al [17] and the European Association for Palliative Care [18]. In a systematic review of physicians’ survival prediction in terminally ill cancer patients, Glare et al evaluated 8 published studies providing 1563 individual prediction-survival dyads. Clinical prediction of survival (CPS) was generally overoptimistic, with the median CPS 42 days and the median actual survival (AS) 29 days. The CPS was correct to within one week in only 25% of cases and overestimated survival by at least four weeks in 27%. The survival of patients was typically 30% shorter than predicted.

The Steering Committee of the European Association for Palliative Care (EAPC) published their evidence-based clinical recommendations on the prognostic factors in advanced cancer patients. In their analysis of the 16 eligible studies, the correlation coefficient of the CPS and AS varied between 0.2 and 0.65. CPS was more than twice as likely to be overoptimistic versus overpessimistic and to overestimate the length of actual survival by a factor of between 3 and 5. The committee recommends clinicians should consider using CPS in combination with other prognostic factors or scores to improve the accuracy of their predictions [18].

The first study investigating a prognostic model in 395 patients contained six significant covariates: primary cancer, site, site of metastases, KPS, fatigue, appetite, and shortness of breath [6]. The discrimination C index for this model was 0.73, 0.75 and 0.81 for discrimination between patients surviving past 3, 6, and 12 months, respectively.

The subsequent study attempted to simplify this six variable model to include only three factors: primary cancer site, site of metastases, and KPS [7]. Utilizing the SPS method, a training set, temporal validation set and external validation set had C indices of 0.66, 0.65 and 0.63, respectively. Using the NRF method, D statistics were listed as 0.65, 0.66, and 0.63 for the same above three sets, respectively. The patient characteristics can be seen in Table 6 for comparison with the current validation set.

**Table 6:** Summary of Patient Characteristics From Two Previous Survival Prediction Investigations

	1999, RRRP (n = 395)	2000, RRRP (n = 445)	2002, PMH (n = 468)
Gender	N (%)	N (%)	N (%)
Male	198 (50%)	243 (56%)	246 (53%)
Female	197 (50%)	202 (45%)	222 (47%)
Age (years)			
Median	68	69	66
Range	31 - 93	24 - 93	24 - 91
Primary cancer site			
Lung	143 (36%)	132 (30%)	266 (55%)
Breast	80 (20%)	99 (22%)	62 (13%)
Prostate	56 (14%)	70 (16%)	24 (5%)
Others	116 (30%)	144 (32%)	126 (27%)
Weight loss			
≥ 10% over the last 6 months	132 (33%)	141 (32%)	108 (23%)
Site of metastases			
Bone only	113 (29%)	164 (37%)	80 (17%)
Others	282 (71%)	281 (63%)	388 (83%)
Karnofsky performance score			
10 - 20, ECOG 4	2 (0.5%)	2 (0.4%)	32 (7%)
30 - 40, ECOG 3	44 (11%)	56 (13%)	112 (24%)
50 - 60, ECOG 2	163 (41%)	208 (47%)	140 (30%)
70 - 80, ECOG 1	167 (42%)	152 (34%)	160 (34%)
90 - 100, ECOG 0	19 (5%)	27 (6%)	23 (5%)
Median	60	60	2
Range	10 - 100	10 - 100	0 - 4

This present analysis is limited by the eligibility criteria of RTOG 9714 allowing only breast/prostate primary tumors and KPS > 40, thus limiting the classification ability of the original model. We have validated the low risk group in SPS model and the low/intermediate risk groups in the NRF model but cannot draw conclusions about the high risk patients. We encourage other investigators to validate especially the high risk groups. Until then, as Parkes encourages, when predictions are needed, physicians should make use of the available validated predictive instruments such as the current one.

Studies have been conducted to determine the optimal way to present medical information to patients and their families. It has been shown that survival curves are a potentially powerful tool to communicate information about health and treatment outcomes [19]. Survival curves provide a graphic presentation of the risk of an outcome over time, as they include a large amount of information that is difficult to convey with numbers alone [20, 21]. Furthermore, use of survival curves avoids the problem of having to select the time points to present–this is important as such a selection has been shown to influence treatment choice [22]. Figure 1 may be of help to clinicians when counseling on survival.

There has been no study examining whether an accurate prediction of survival can improve actual clinical care, nor investigating if the models improve the decision making in the care of this group of patients. Future studies should also evaluate the inception criteria and define common inception points for the accrual of patients in studies of advanced disease. Quality of life assessment including self-rated health may assist in the selection of homogeneous cohorts of patients with terminal cancer and fine-tune the prognostic models.
